# Synthesis and Theoretical Study of Molecularly Imprinted Nanospheres for Recognition of Tocopherols

**DOI:** 10.3390/molecules14082985

**Published:** 2009-08-12

**Authors:** Theeraphon Piacham, Chanin Nantasenamat, Thummaruk Suksrichavalit, Charoenchai Puttipanyalears, Tippawan Pissawong, Supanee Maneewas, Chartchalerm Isarankura-Na-Ayudhya, Virapong Prachayasittikul

**Affiliations:** Department of Clinical Microbiology, Faculty of Medical Technology, Mahidol University, Bangkok 10700, Thailand

**Keywords:** tocopherol, vitamin E, molecular imprinting, molecularly imprinted polymer, MIP, molecular modeling

## Abstract

Molecular imprinting is a technology that facilitates the production of artificial receptors toward compounds of interest. The molecularly imprinted polymers act as artificial antibodies, artificial receptors, or artificial enzymes with the added benefit over their biological counterparts of being highly durable. In this study, we prepared molecularly imprinted polymers for the purpose of binding specifically to tocopherol (vitamin E) and its derivative, tocopherol acetate. Binding of the imprinted polymers to the template was found to be two times greater than that of the control, non-imprinted polymers, when using only 10 mg of polymers. Optimization of the rebinding solvent indicated that ethanol-water at a molar ratio of 6:4 (v/v) was the best solvent system as it enhanced the rebinding performance of the imprinted polymers toward both tocopherol and tocopherol acetate with a binding capacity of approximately 2 mg/g of polymer. Furthermore, imprinted nanospheres against tocopherol was successfully prepared by precipitation polymerization with ethanol-water at a molar ratio of 8:2 (v/v) as the optimal rebinding solvent. Computer simulation was also performed to provide mechanistic insights on the binding mode of template-monomer complexes. Such polymers show high potential for industrial and medical applications, particularly for selective separation of tocopherol and derivatives.

## 1. Introduction

Reactive oxygen species are produced during normal aerobic metabolism and are eliminated by antioxidative enzymes and compounds. Perturbation to this equilibrium triggers a condition known as oxidative stress that has been associated with a wide range of diseases. Vitamin E, commonly known as a-tocopherol (TP), has attracted much interest in recent years due to its multifaceted therapeutic potential. Many reports have suggested that supplementation with vitamin E may help reduce the risk of cardiovascular diseases [1], cancer [2], and neurodegenerative diseases such as Alzheimer’s disease. Mechanistically, tocopherol functions *in vivo* as a potent peroxyl radical scavenger which displays an ability to competitively bind to peroxyl radical a thousand-fold greater than that of polyunsaturated fatty acids [3] by donating its electrons to stabilize the radicals.

Tocopherol is found abundantly in vegetables and fruits [4,5]. Because of its high medical importance, it is desirable to develop ways to rapidly and accurately assess the concentrations of vitamin E in foodstuffs. The gold standard approach for determination of vitamin E relies on chromatographic methods such as high performance liquid chromatography, supercritical fluid chromatography, capillary gas chromatography, and thin layer chromatography. The drawback of such approaches is that they are labor-intensive and require tedious sample pre-treatment such as saponification [6], transesterification [7], distillation, solvent extraction, membrane separation [8], crystallization, and supercritical CO_2_ extraction [9].

**Figure 1:**
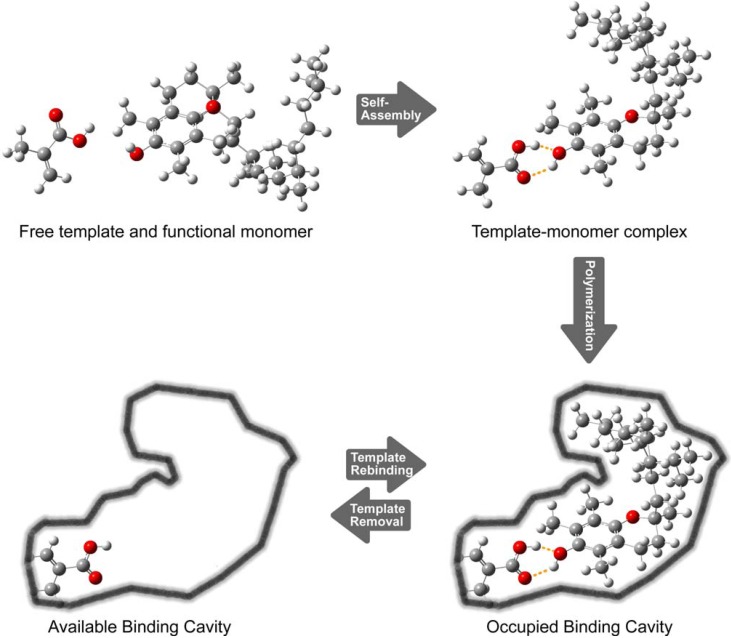
Schematic representation of the molecular imprinting process.

Molecular imprinting is a simple technique for preparing tailor-made affinity adsorbents possessing specific binding sites within polymer matrices [10,11]. The molecular imprinting process, as shown in Figure 1, essentially involves three main steps: (i) self-assembly of template and functional monomer molecules, (ii) polymerization of template-monomer complex with cross-linking monomers, and (iii) template removal to unveil binding cavity that is specific to the imprint molecule. 

The molecularly imprinted polymers (MIPs) have been demonstrated to possess excellent properties for separation of many interesting compounds, ranging from small molecules to macromolecules [12,13,14]. Moreover, MIPs can bind specifically to their original and related templates, and possess tolerance to mechanical stress, temperature, pH, acid-base, etc. Owing to their robust properties, MIPs are suitable for broad range of applications as separation media for chromatography [15] and solid phase extraction [16], nanoreactors for combinatorial synthesis of novel enzyme inhibitors [17], recognition elements for biosensors [14,18], artiﬁcial receptors for drug assays [19], synthetic receptors for peptides [20] and biological molecules [21,22], biological receptor mimics [23], drug delivery [24] and enzyme mimetics [25,26,27].

In this study, we explore the utilization of MIPs as recognition unit for tocopherol and its derivative tocopherol acetate (TPA) using methacrylic acid as functional monomer, dicholoromethane and acetonitrile as porogenic solvents. The first part of the study focused on improving upon the binding performance of bulk polymers prepared by thermal polymerization. The second portion of the study discusses for the first time the preparation of nanospheres molecularly imprinted toward tocopherols via precipitation polymerization. Molecular modeling was then used to analyze and discern the relative strength of molecular interaction between the template molecules and functional monomers. The produced polymers exhibited good prospects for future application as robust separation matrices for the purification of tocopherols and derivatives.

## 2. Results and Discussion

### 2.1. Preparation of tocopherol-imprinted polymers

**Figure 2:**
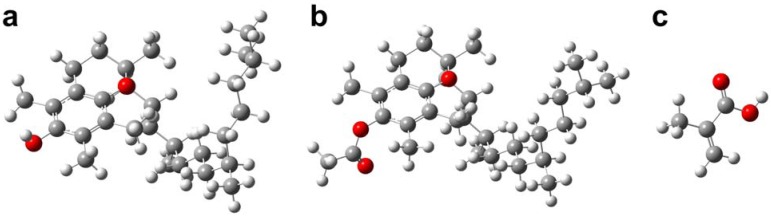
Molecular structures of tocopherol (a) and tocopherol acetate (b) and methacrylic acid (c).

The first report of TP-imprinted polymers utilized the traditional bulk polymerization method, which had yielded imprinted polymers with rather high non-specificity when compared to the non-imprinted polymers [28]. In light of this, a series of works have been published on the molecular imprinting of TP in efforts to enhance its binding performance. Such approaches utilize supramolecules such as calixarenes as functional monomer [29] or by the semi-covalent approach where the functional monomers are covalently linked to the template molecule in the pre-polymerization step subsequently followed by base hydrolysis to remove the template molecule [30]. Novel applications of MIPs as drug delivering materials have also been demonstrated by Puoci and co-workers [31]. This study reports the preparation of TP-imprinted polymers using higher cross-linking density and more polar porogenic solvent via thermal-induced bulk polymerization method as well as the preparation of TP-imprinted nanospheres via precipitation polymerization. The pre-polymerization mixture was prepared using methacrylic acid as functional monomer and ethylene glycol dimethacrylate as cross-linking monomer. These components were solubilized in dichloromethane and acetonitrile for preparation of monolithic bulk polymers and nanospheres, respectively. The molecular structures of the imprint molecules and functional monomer are shown in Figure 2 as ball-and-stick model.

### 2.2. Recognition properties of tocopherol-imprinted polymers

**Figure 3:**
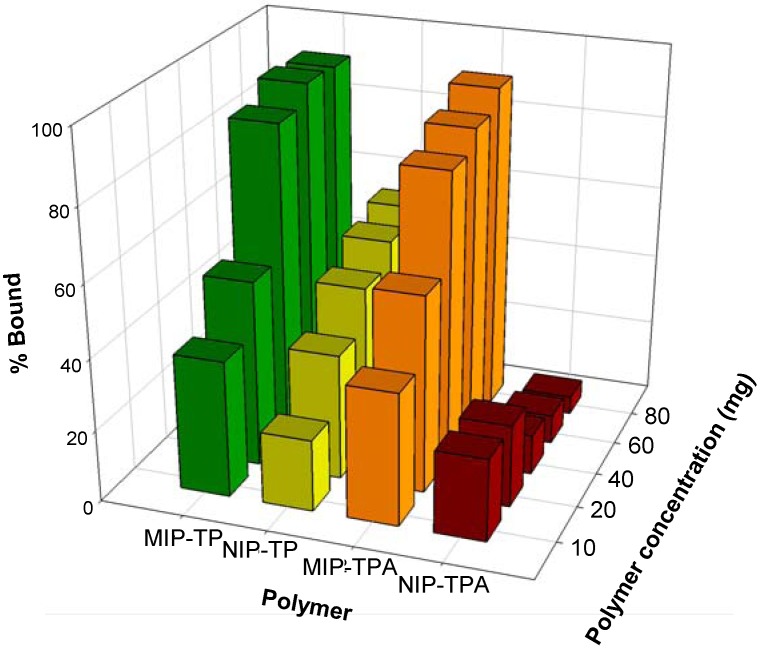
Rebinding analysis of imprinted and non-imprinted polymers towards its respective template molecule.

Preliminary rebinding analysis was performed in the porogenic solvent dichloromethane in which the polymer was formed. It was observed (data not shown) that the solvent could not provide distinctive differences in the binding performance of imprinted and non-imprinted polymer. This is in agreement with the previous report that the solvent used for polymerization did not afford good rebinding performance [28]. Owing to the amphipathic properties of tocopherol in that it is comprised of both hydrophobic and hydrophilic moieties, the desired rebinding solvent should be able to solubilize the tocopherols, while at the same time minimize interaction with the polar aqueous solution which competes with the functional monomers in binding to the template molecules. To meet such requirements, the selected solvents used for rebinding are binary mixtures of ethanol with water at a molar ratio of 6:4 v/v. As shown in Figure 3, both TP- and TPA-imprinted polymers displayed selective recognition towards the template molecules TP and TPA as compared to the respective non-imprinted polymers. It is observed that at 10 mg of polymer, both TP and TPA imprinted polymers displayed two-fold greater binding capacity towards the template than the corresponding non-imprinted polymers. The calculated binding capacity of all imprinted polymers was approximately 2 mg/g of polymer. Upon increasing the polymer concentration, both of the imprinted polymers exhibited higher binding capacities with high non-specific binding afforded by the TP imprinted polymers. On the other hand, the non-imprinted polymers displayed quite low non-specificity towards the template molecule. The better selectivity afforded by the TPA imprinted polymer can be ascribed to the differences in the binding capacity between imprinted and non-imprinted polymers which were significantly greater for TPA imprinted polymer. Possible explanation for such observation can be attributed to the fact that TPA can act as only a hydrogen bond acceptor whereas the dual properties of TP allows it to act as both hydrogen bond donor and acceptor, which therefore predisposes it to higher non-specific binding with the non-imprinted polymers.

In this study, the monolithic polymers were prepared by thermal-induced polymerization which was similar to the initial efforts of Puoci *et al*. [28] The bulk polymers developed herein had the following notable differences with the previous report: higher cross-linking density and more polar porogenic solvent. Comparison in the binding performance of the polymers prepared in this study and that reported previously, has an imprinting effect of 2 for the former, while for the latter no imprinting effect was observed for thermal-induced polymerization and imprinting effect of 1.47 was observed for photo-induced polymerization of the imprinted polymers in relation to the non-imprinted polymers as demonstrated in this paper. Discrepancies in the binding performances of the MIPs reported in this study and that previously reported could be attributed to differences in the degree of cross-linking density as the molar ratio of template:monomer:cross-linker used in this study was 0.5:8:50 whereas the previously reported ratio was in the range of 1:8:25 to 1:16:25. Such variation in the cross-linking density may give rise to different degree of rigidity of the polymers suggesting that polymers with more rigidity would exert better binding performance. Another possible explanation for the higher imprinting effect of polymers described in this paper may also be attributed to differences in the chemical properties of the porogenic solvents used in this study and that previously reported. In this regard, the polymers described herein were prepared in dichloromethane (dielectric constant of 8.93) while those previous reported were made in chloroform (dielectric constant of 4.8069). On the basis of the dielectric constant, dichloromethane is clearly a more polar solvent than chloroform, which is also more favorable for subsequent rebinding in the polar binary mixture of ethanol (dielectric constant of 25.3) and water (dielectric constant of 80.1). An added benefit of the use of more polar porogenic solvent is that it helps reduce the polarity gap of solvent used for polymer preparation and solvent used for rebinding experiments. Such reduction in the polarity gap helps reduce the degree of polymer swelling, which may perturb the binding cavity and adversely affect the binding performance. 

**Figure 4:**
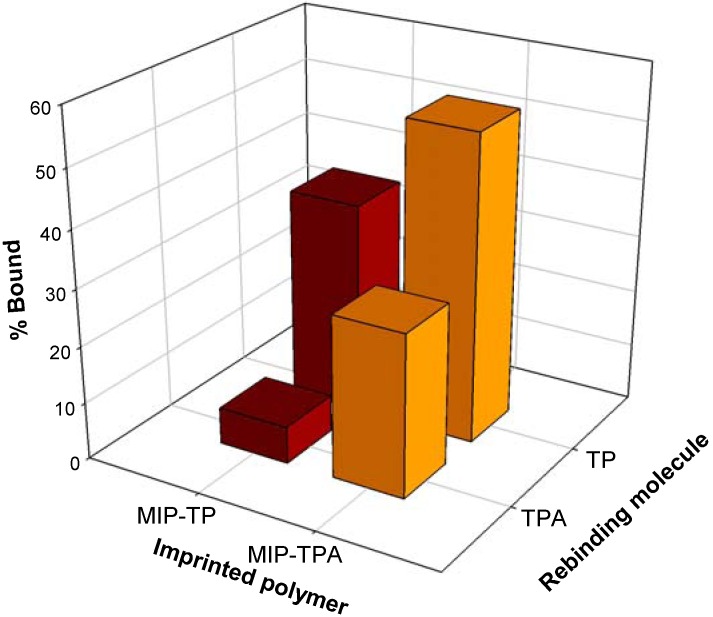
Cross-selectivity of MIP-TP and MIP-TPA with templates TP and TPA.

To determine the cross-selectivity of the prepared polymers, both imprinted polymers were cross-bound with the two template molecules using 40 mg of polymer that was incubated in 1 mL of each template (50 μg mL^-1^) solubilized in a solution of ethanol:water (6:4, v/v). The cross-selectivity results as represented in Figure 4 show that the TP imprinted polymer can bind to the TP molecules (37.2%) significantly greater than the competing TPA molecules (6.5%). However, the TPA imprinted polymer can bind to the TPA molecules (28.6%) at approximately half of the competing TP molecules (53.8%). A plausible explanation for such observation is attributed to differences in the molecular structures of TP and TPA where the former is smaller than the latter, particularly, the former has a hydroxyl moiety whereas the latter possesses a more bulky acetate ester group. Consequently, the imprinted cavity for TPA imprinted polymers would likewise be larger than that of the TP imprinted polymers, which would allow the smaller TP molecules to easily occupy the binding cavity within the macromolecular matrices of TPA imprinted polymers. On the other hand, the smaller imprinted cavity of TP imprinted polymers would not accommodate the much larger TPA molecules.

**Figure 5:**
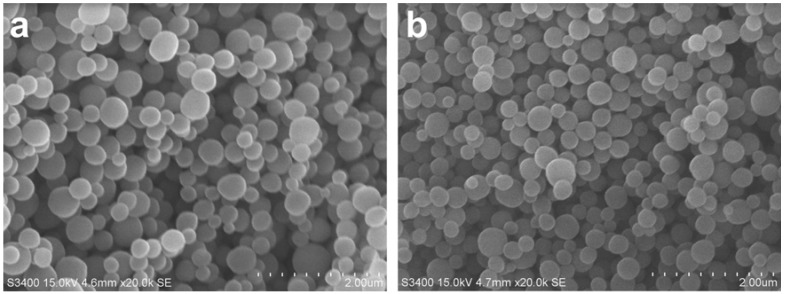
SEM micrograph of TP-imprinted nanospheres (a) and non-imprinted nanospheres (b).

Aside from the preparation of imprinted bulk monoliths, we had also molecularly imprinted nanospheres towards tocopherol (MIN-TP) via the precipitation polymerization approach. The morphology of the molecularly imprinted nanospheres was characterized by scanning electron microscope (SEM) as shown in Figure 5 to be uniform in size and shape. The nanospheres were spherical and the particle size was estimated to be in the range of 200–400 nm. 

**Figure 6:**
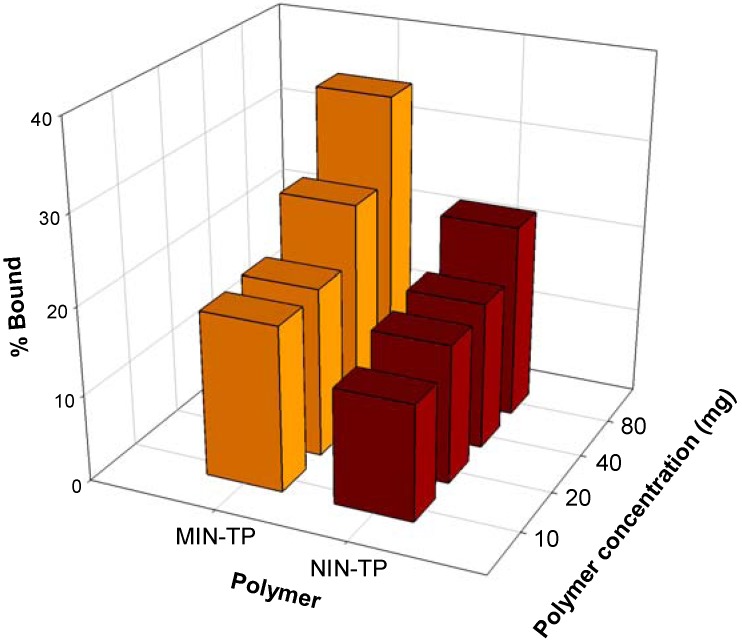
Rebinding analysis of TP-imprinted and non-imprinted nanospheres with TP.

Rebinding analysis of the imprinted nanospheres was determined by batch mode analysis and its binding performance is indicated in Figure 6. Results indicated that the imprinted nanospheres were able to bind selectively to TP as observed from the imprinting effect, which can be inferred from differences in the binding capacity of imprinted and non-imprinted nanospheres in the range of 1.22–1.57-fold for polymers with concentrations of 10–80 mg mL^-1^. Polymer concentrations of 40 and 80 mg mL^-1^ had the highest differences at 1.51 and 1.57 fold, respectively, with binding capacity of 25.55 and 16.94 % for imprinted and non-imprinted polymers at 40 mg mL^-1^ and binding capacity of 34.84 and 22.24 % at 80 mg mL^-1^.

**Figure 7:**
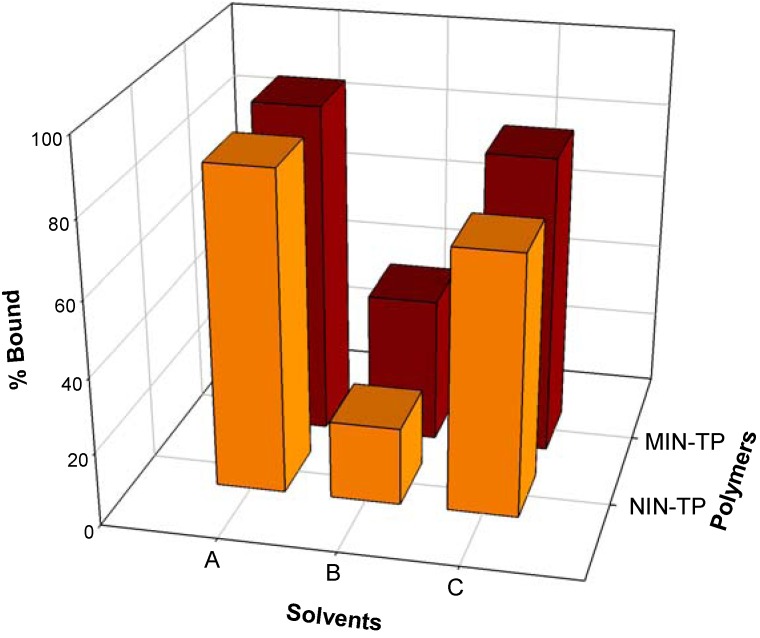
Rebinding of TP-imprinted and non-imprinted nanospheres with TP in various solvent systems: acetonitrile:water (1:1, v/v) (A), ethanol:water (8:2, v/v) (B), ethanol:water (6:4, v/v) (C).

To maximize the binding capacity of the prepared nanospheres, the optimal rebinding solvent was empirically determined using binary mixtures of either acetonitrile with water or ethanol with water [32]. Fixed amount (40 mg) of MIN or NIN was tested with 0.1 mg mL^-1^ of TP in different types of solvent, which included acetonitrile:water (1:1, v/v), ethanol:water (8:2, v/v) and ethanol:water (6:4, v/v). The result of this solvent optimization is shown in Figure 7 where the appropriate solvent was identified to be ethanol:water (8:2,v/v) as it afforded the highest imprinting effect of 1.85 with a binding capacity of 39 and 21%, respectively, for imprinted and non-imprinted nanospheres.

It is observed that the binding performances of both molecularly imprinted bulk polymers and nanospheres are discretely different in that the former gave higher binding capacity. In spite of this, the imprinting effect of the nanospheres was comparable to that of the bulk polymers. Nevertheless, the potential advantages of the imprinted nanospheres far outweight its binding capacity shortcoming in the following respect when compared to bulk polymers: (i) monodisperse particles [33], (ii) precise control over the polymerization process [34], (iii) colloidal stability [35], (iv) larger surface area [34], (v) homogeneous binding sites [33], (vi) higher association constants [36], (vii) faster mass transfer kinetics [37], and (viii) faster binding kinetics [38]. In practical terms, the monodisperse nanospheres are more suited for applications in separation as they are known to pack efficiently in chromatograhic columns by providing good flow properties, low back pressure, and good column efficiency. In regards to the traditional approach of MIP preparation by bulk polymerization, the synthesized monolithic polymer possess the following undesirable properties which limit its scope of application: (i) irregular particles, (ii) limited control over the polymerization process [39], (iii) low yield and time-consuming [40], (iv) heterogeneous binding sites [36], and (v) poorly accessible binding sites [41]. Owing to the irregularity in size and shape of the bulk polymers, the irregular particles have poor chromatography efficiency and are therefore unsuited to serve as biorecognition elements in novel applications as MIP-based assays or MIP-based sensor arrays [34].

### 2.3. Molecular modeling of template-monomer complex

Nicholls and colleagues [42] previously pointed out in their thermodynamics study that one of the major factors governing the binding performance of MIPs lies in the relative strengths of the template-monomer complexes. Therefore, to gain mechanistic insight into the binding performance of the imprinted polymers, computer simulation was performed to discern the relative strengths of interaction between TP or TPA with MAA. The usefulness of computational approaches for elucidating and modeling the interaction strengths of MIPs had previously been demonstrated in our previous investigations [43,44,45,46] on MIP systems. These included the neural network method pioneered by our group for quantitatively correlating the structures of template molecules and functional monomers with their respective imprinting factor values [44,45,46], an approach which had been successfully applied for modeling a wide range of molecular systems [47,48,49,50,51]. A practical overview of this quantitative structure-property relationship paradigm is provided in a recent review [52]. The methodology used herein is essentially based on theoretical calculations of the interaction energy for putative template-monomer adducts. The relative strength of a given template-monomer adduct can be inferred from calculated interaction energy whereby high interaction energy implied strong association between the template and functional monomers. Such approach has previously been demonstrated to be useful in the selection of promising functional monomers from a vast library of compounds. For this study, we utilize this computational method for retrospectively analyze and elucidate the mechanistic details and mode of interaction for the TP-MAA and TPA-MAA adducts. The interaction energy is calculated by first deriving the geometrically optimized structures at the Hartree-Fock level of theory in combination with the 3-21g(d) basis sets followed by single-point calculation at the density functional theory level using the B3LYP functional in combination with 6-31g(d) basis set. 

The quantum chemical calculations were performed on possible modes of interaction for the pre-polymerization complexes. It is apparent that the template-monomer complexes can exist in various combinations of conformers, therefore simplified models of the template-monomer complexes existing at 1:1 ratio were used for the computational study. The molecular models were derived by manually docking the functional monomer to each functional group of the template molecule. A summary of the molecular properties for the sampled conformations of the template-monomer complex are provided in Table 1. 

**Table 1:** Summary of the interaction energies of template-monomer complexes.

	*E* (a.u.)	Δ *E* (a.u.)^ a^	Δ *E* (kJ mol^-1^)^ b^
TP	–1285.682		
TPA	–1438.350		
MAA	–306.475		
TP–MAA(1)	–1592.180	–0.023	–61.042
TP–MAA(2)	–1592.167	–0.010	–27.159
TP–MAA(3)	–1592.172	–0.015	–40.222
TPA–MAA(1)	–1744.844	–0.020	–51.514
TPA–MAA(2)	–1744.840	–0.015	–39.900
TPA–MAA(3)	–1744.839	–0.015	–38.108

^a^Δ*E* is the interaction energy calculated from Δ*E* = *E*_template-monomer_ – *E*_template_ – *E*_monomer_ ^b^Δ*E* is converted from a.u. to kJ mol^-1^ using the conversion factor 2.626 × 10^3^.

**Figure 8:**
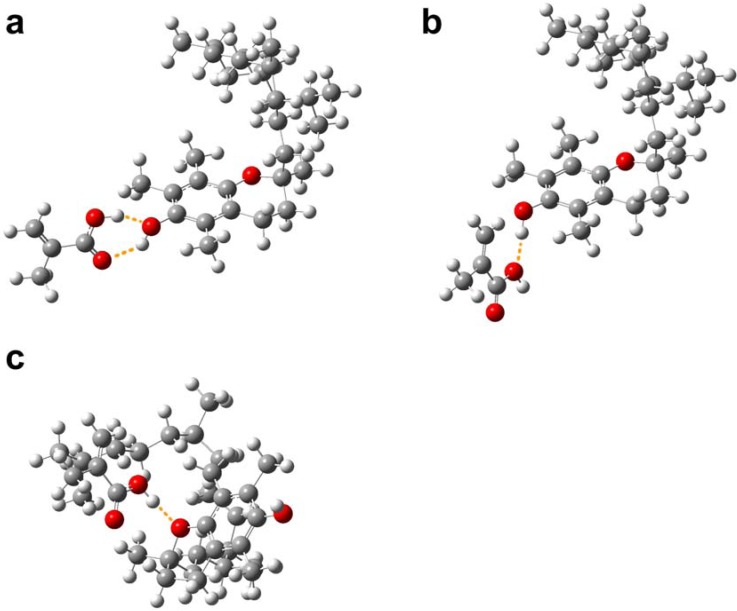
Possible modes of interaction of TP with MAA.

A total of 3 possible conformers for TP–MAA complexes were obtained from the molecular simulation as shown in Figure 8. As observed in Figure 8a, the carboxylic acid moiety of MAA could interact with the hydroxyl moiety of TP in a two point interaction, which yielded the highest interaction energy of –61.042 kJ mol^-1^. Figure 8b demonstrates a one-point interaction of MAA’s hydroxyl oxygen with TP’s hydroxyl hydrogen, which gave significantly lower interaction energy of–27.159 kJ mol^-1. A third possible TP–MAA conformer is shown to utilize MAA’s hydroxyl hydrogen to interact with TP’s ether oxygen. Such complex gave the second highest interaction energy in this set with a value of –40.222 kJ mol-1^.

**Figure 9:**
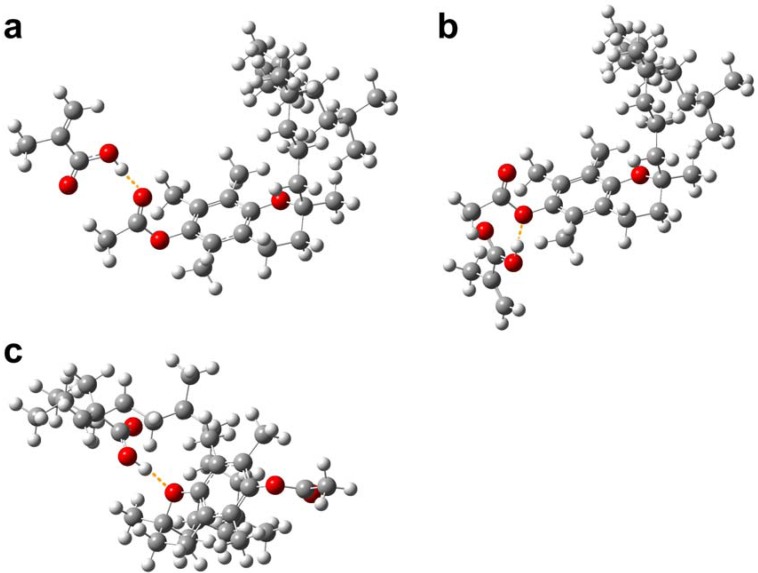
Possible modes of interaction of TPA with MAA.

The possible conformations for TPA–MAA complexes are illustrated in Figure 9. It can be seen in Figure 9a that MAA can act as a hydrogen bond donor by using the hydrogen atom of the hydroxyl group to engage in one point interaction with the carbonyl oxygen of the terminal ester group with interaction energy of –51.514 kJ mol^-1^. Figure 9b illustrates the interaction of MAA’s hydroxyl hydrogen with TPA’s ester oxygen in a one point interaction with an energy of –39.900 kJ mol^-1^. Figure 9c shows the interaction of MAA’s hydroxyl hydrogen with TPA’s central ether oxygen with an interaction energy of –38.108 kJ mol^-1^. It should be noted that TPA can only act as a hydrogen bond acceptor due to absence of the hydrogen atom and can therefore engage in only one point of interaction with MAA. This is unlike TP which possesses both hydrogen bond acceptor and donor and can therefore interact with MAA at two points of interaction thereby affording higher strengths of interaction.

Molecular descriptors that are commonly used for elucidating the chemical properties of molecules in terms of its stability and reactivity [53] included the energy of the highest occupied molecular orbital (HOMO), the energy of the lowest unoccupied molecular orbital (LUMO), and the energy difference of HOMO and LUMO which is also known as the HOMO–LUMO gap. The former represents the electron-donating ability while the latter represents the electron-withdrawing ability of the molecules. Furthermore, the energy differences between the HOMO and LUMO level represent the stability and chemical reactivity of a molecule, where large values indicate high molecular stability and low chemical reactivity while small values give rise to low molecular stability and high chemical reactivity. Therefore, the HOMO–LUMO gap can be used as a relative index for the degree of interaction strength between templates and monomers in which lower values indicate higher strengths of interaction. According to this notion, the molecular interaction between the template and functional monomer (in the range of 3.826-4.911 eV for TP–MAA and 4.716–5.181 eV for TPA–MAA) caused a marked decrease in the HOMO–LUMO energy gap (as shown in Table 2) when compared to the free forms of the template (6.299 and 5.717 eV for TP and TPA, respectively) and functional monomer (5.728 eV for MAA). Furthermore, it is observed that TP–MAA (3.826-4.911 eV) possessed lower HOMO–LUMO gap than that of TPA–MAA (4.716–5.181 eV), which in addition to the calculated interaction energies, suggests that the molecular interaction of TP–MAA complex was higher than that of TPA–MAA.

Although the experimental results may suggest that TPA-imprinted polymer provided better imprinting effects (as observed by the difference in binding performance of imprinted and non-imprinted polymers) than that of TP-imprinted polymer. However, the former did not afford good cross-selectivity as it can cross-bind to TP at significantly higher capacity than that of the imprint molecule. On the other hand, although TP-imprinted polymers may provide lower imprinting effect than the TPA-imprinted polymers, it offsets this by maintaining stellar binding performance towards only the imprint molecule while not being able to cross-bind to TPA. 

Such observations could be accounted for by the mechanistic insights provided by the computer simulations. Firstly, the lower imprinting effect of TP-imprinted polymers could be explained by the fact that TP can interact more strongly with MAAs and that TP can engage in more points of interaction in that it can act as both hydrogen bond acceptor and donor. Following this notion, TP would also be expected to interact non-specifically with the randomly oriented MAAs in the macromolecular matrices of the non-imprinted polymers. On the other hand, as TPA can act as only a hydrogen bond acceptor it can likewise engage in a weaker interaction with the MAA which also decreased its chances of interaction with the randomly oriented MAAs of the non-imprinted polymers. Secondly, the better cross-selectivity provided by TP-imprinted polymers is due to the fact that TPA is a larger molecule which would find it difficult to transfer through the somewhat smaller and restricted cavity of the macromolecular matrices of the imprinted polymers. As a result, the TP-imprinted polymers would allow only TP to enter while restricting access to TPA as a result of the difficulty in mass transfer through the polymer. The opposite applies to the TPA-imprinted polymers where TP, which has a molecular weight of 430.706 g/mol, can easily enter the macromolecular matrices of the imprinted polymers as it is significantly smaller than the TPA molecules, which has a molecular weight of 472.743 g/mol.

**Table 2:** Summary of the quantum chemical parameters of template-monomer complexes.

	*E*_HOMO_	*E*_LUMO_	*E*_HOMO–_ _LUMO_
TP	–7.442	–1.143	6.299
TPA	–5.371	0.346	5.717
MAA	–5.492	0.236	5.728
TP–MAA(1)	–5.446	–1.032	4.414
TP–MAA(2)	–5.184	–1.359	3.826
TP–MAA(3)	–5.749	–0.839	4.911
TPA–MAA(1)	–5.664	–0.604	5.059
TPA–MAA(2)	–5.646	–0.930	4.716
TPA–MAA(3)	–5.959	–0.778	5.181

The energies of HOMO, LUMO, and their gaps were converted from a.u. to eV using the conversion factor of 27.2114.

## 3. Conclusions

In conclusion, we have successfully prepared molecularly imprinted polymers as bulk monoliths and nanospheres for selective recognition of tocopherol and tocopherol acetate. The mechanistic insights into the binding modes of the template-monomer complexes were elucidated from computer simulation and their results were well correlated with the experimental results. The rebinding of the prepared polymers in various concentrations of ethanol-water binary mixtures was performed to discern the optimal aqueous-based rebinding solution. The MIPs described herein has great potential for future application in separation and extraction of tocopherols from biological milieu both at the academic and industrial settings.

## 4. Experimental

### 4.1. Chemicals

Tocopherol (TP) and tocopherol acetate (TPA), methacrylic acid (MAA), ethylene glycol dimethacrylate (EDMA), 2,2’-azoisobutyronitrile (AIBN) were purchased from Sigma-Aldrich. Dicholoromethane (DCM) and acetonitrile was purchased from Merck. All solvents were of analytical or HPLC grade. 

### 4.2. Preparation of molecularly imprinted polymers

Molecularly imprinted polymers were prepared in DCM using TP or TPA as template molecule and MAA as functional monomer. TP or TPA (0.5 mmol) was dissolved in DCM (10 mL) containing MAA (8 mmol). To the solution was then added the crosslinker, EDMA (50 mmol), and the initiator, AIBN (202 mg). The obtained solution was transferred into a 20 mL screw-capped borosilicate tube and purged with argon for 10 min. The tube was then submerged in a 60 °C water bath for 18 h. The solid polymer was smashed and ground with a mechanical mortar. Particles with apparent diameter of 10-25 μm were collected by repetitive sieving and sedimentation in acetone. To remove the template, methanol containing 15% acetic acid (v/v) was used for extraction. Quantitative removal of the template was ensured by monitoring the amount of template remaining in the extraction solvent by UV spectrophotometry. The non-imprinted control polymers were prepared in a similar manner as used for the corresponding imprinted polymers except for omission of the template molecule during polymerization.

### 4.3. Preparation of molecularly imprinted nanospheres

Molecularly imprinted nanosphere was prepared via precipitation polymerization [54] in acetonitrile using tocopherol as template molecule. The contents of template and monomer were the same as those prepared by bulk polymerization except for the use of excess solvent which was increased by 20-fold. The template molecules were directly removed from the produced nanospheres by Soxhlet extraction using methanol containing 15% acetic acid (v/v). The non-imprinted control nanospheres were prepared in the same way as that of the corresponding imprinted nanospheres, except for the omission of the template molecule during polymerization.

### 4.4. Scanning Electron Microscopy (SEM)

The particle sizes of the imprinted and non-imprinted nanospheres were determined by scanning electron microscope (HITACHI S-3400). Briefly, the nanospheres were mounted on metallic studs via double sided conductive tape and subsequently applied gold ion coating using sputter coater (Bal-tec SCD 050) for 90 s under vacuum at current intensity of 60 mA and scanning accelerating voltage of 15 kV.

### 4.5. Binding analysis

Binding analysis was carried out by incubating varying amounts of polymer in 1 mL volume of analyte solution (0.1 mg/mL) on a rocking table for 12 h at room temperature. After incubation, the samples were centrifuged at 12,000 rpm for 10 min, from which 0.75 mL supernatant was collected for determination of the free analyte by spectrophotometry at wavelengths of 292 and 283 nm for TP and TPA, respectively.

### 4.6. Molecular modeling analysis

The molecular models of the template molecules, functional monomer and their complexations were drawn using GaussView, version 3.09, and subjected to full geometry optimization without symmetry constraints under Gaussian 03W at the Hartree-Fock level of theory in combination with the 3-21g(d) basis set. The possible modes of interaction between template molecules and functional monomers at molar ratio of 1:1 were sampled by manually docking the functional monomer to each functional group of the template molecule in a systematic manner. The interaction energy of the template-monomer complex as derived at the B3LYP/6-31g(d) level was calculated according to the following equation:

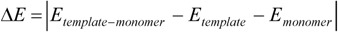
(1)
where 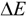 represents the interaction energy, 
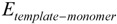
 represents the energy of template-monomer complex, 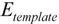 represents the energy of template molecule, and 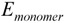 is the energy of functional monomer molecules.
